# Supplementary material for: Investigating the complexity of the double distance problems
Source: Algorithms Mol Biol. 2024 Jan 4;19:1. doi: 10.1186/s13015-023-00246-y (PMC10765962; doi:10.1186/s13015-023-00246-y)
Supplement: Supplementary file 1 — Additional file 1. This file contains additional figures for supporting the proofs of statements in this manuscript. [file 13015_2023_246_MOESM1_ESM.pdf]

Additional File 1 -
Investigating the complexity of the double distance problems

Marília D. V. Braga, Leonie R. Brockmann, Katharina Klerx and Jens Stoye
Faculty of Technology and Center for Biotechnology (CeBiTec), Bielefeld University, Germany

(A) Additional file figures

All the figures presented here assume a graph free of symmetric squares and triplets. For each case we have the ambiguous component of the pruned graph and its intersection graph. Often small modifications (e.g., by switching the positions of $\mathbb{S}$- and $\check{\mathbb{D}}$-telomeres) lead to equivalent cases, and here we show only one of these. In the particular situations of an intersection between two cycles being a $\check{\mathbb{D}}\mathbb{S}\check{\mathbb{D}}$-path or intersections between two paths occurring at both telomeres, the respective vertices of the intersection graph are connected by two parallel edges.

(A).1 Complex bubbles are limited to 8 cycles

By a complete enumeration of cases, in Figures A1-A6 we show that, if a bubble is not a line, it reaches its "capacity" with at most 8 cycles. In all figures dashed gray edges are pruned out.

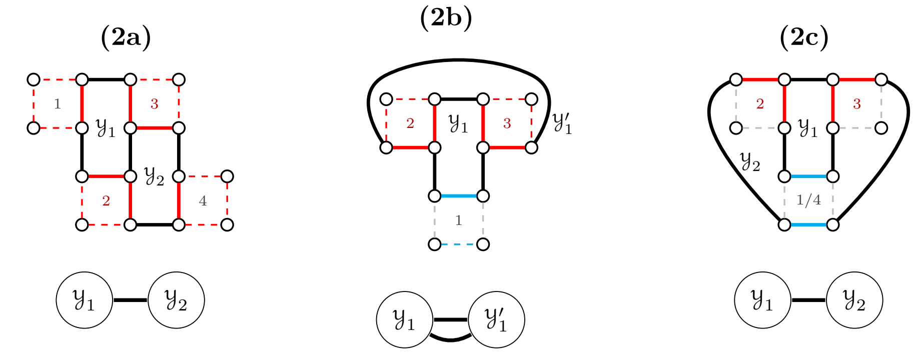

Figure S1 Bubbles with two cycles. (2a) A typical cycle-line. (2b) $\mathcal{Y}_1$ and $\mathcal{Y}_1'$ intersect in a $\check{\mathbb{D}}\mathbb{S}\check{\mathbb{D}}$-path. (2c) is derived from (2a) by merging squares.

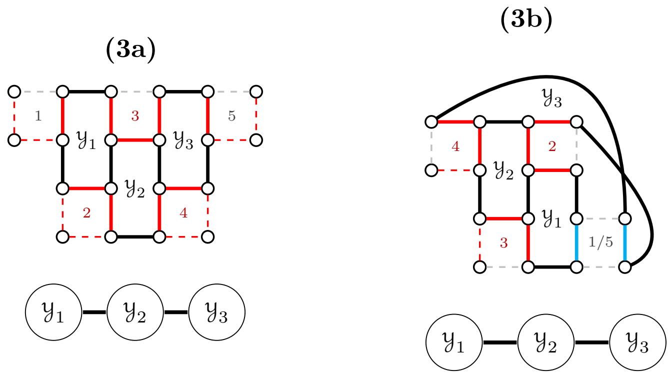

Figure S2 Bubbles with three cycles. (3a) Cycle-line of length 3. (3b) is derived from (3a) by merging squares.

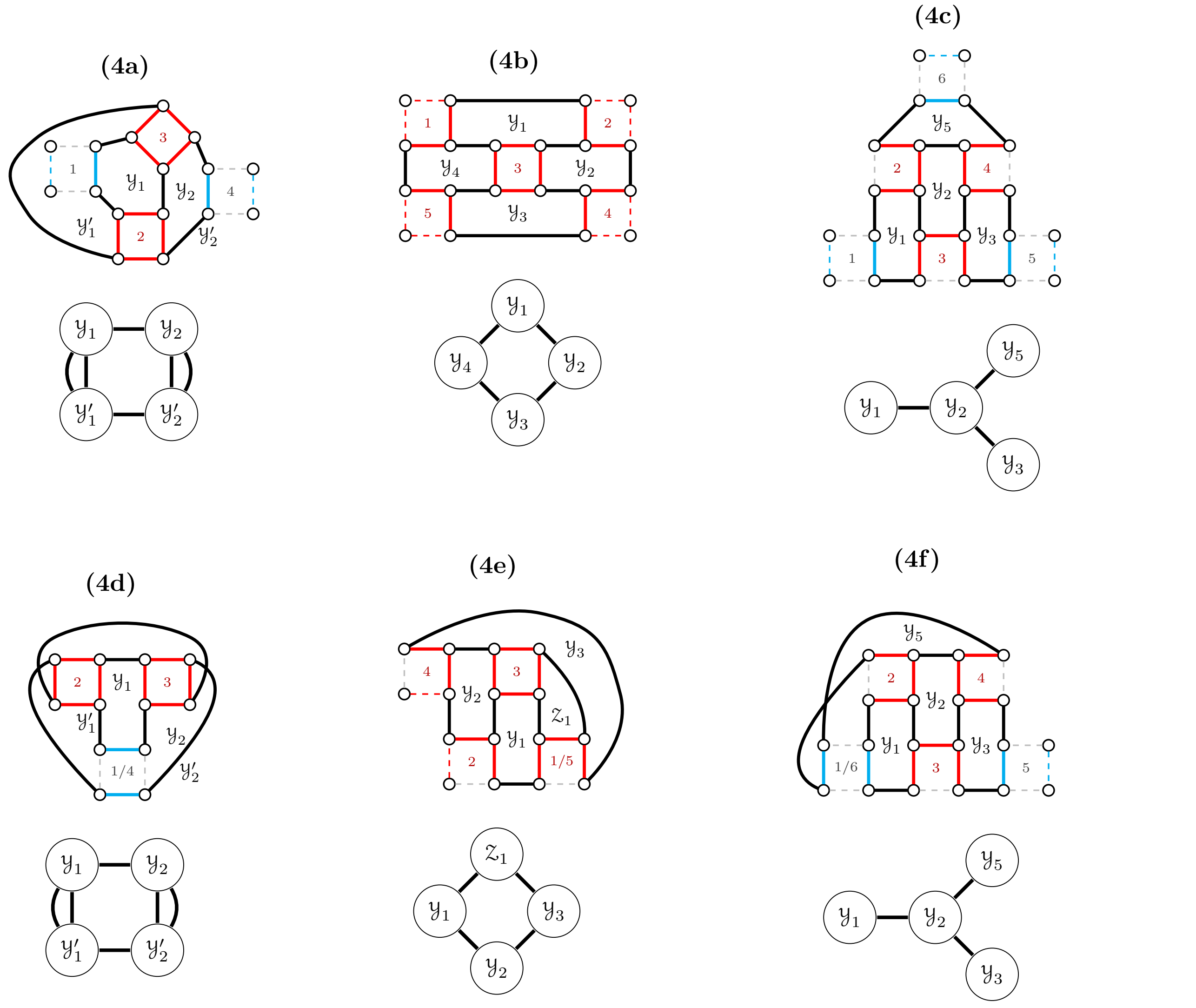

Figure S3 Bubbles with four cycles. (4a) is derived from (2a) by adding a $\check{\mathbb{D}}$-edge or from (2b) by adding a $\check{\mathbb{D}}\mathbb{S}\check{\mathbb{D}}$-path. (4b) is derived from (3a) by adding a $\check{\mathbb{D}}$-edge. (4c) is derived from (3a) by adding a $\check{\mathbb{D}}\mathbb{S}\check{\mathbb{D}}$-path. (4d) is derived from (4a) by merging two squares. (4e) is derived from (3a) by merging two squares (this gives an additional 4-cycle). (4f) is derived from (4c) by merging two squares.

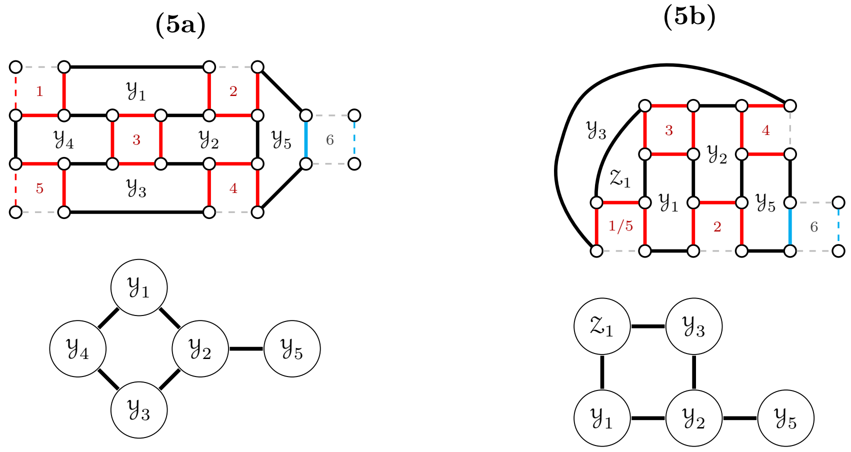

Figure S4 Bubbles with five cycles. (5a) is derived from (4b) by adding a $\check{\mathbb{D}}$-edge or from (4c) by adding a $\check{\mathbb{D}}\mathbb{S}\check{\mathbb{D}}$-path. (5b) is derived from (4c) by merging two squares (this gives an additional 4-cycle) or from (4e) by adding a $\check{\mathbb{D}}\mathbb{S}\check{\mathbb{D}}$-path.

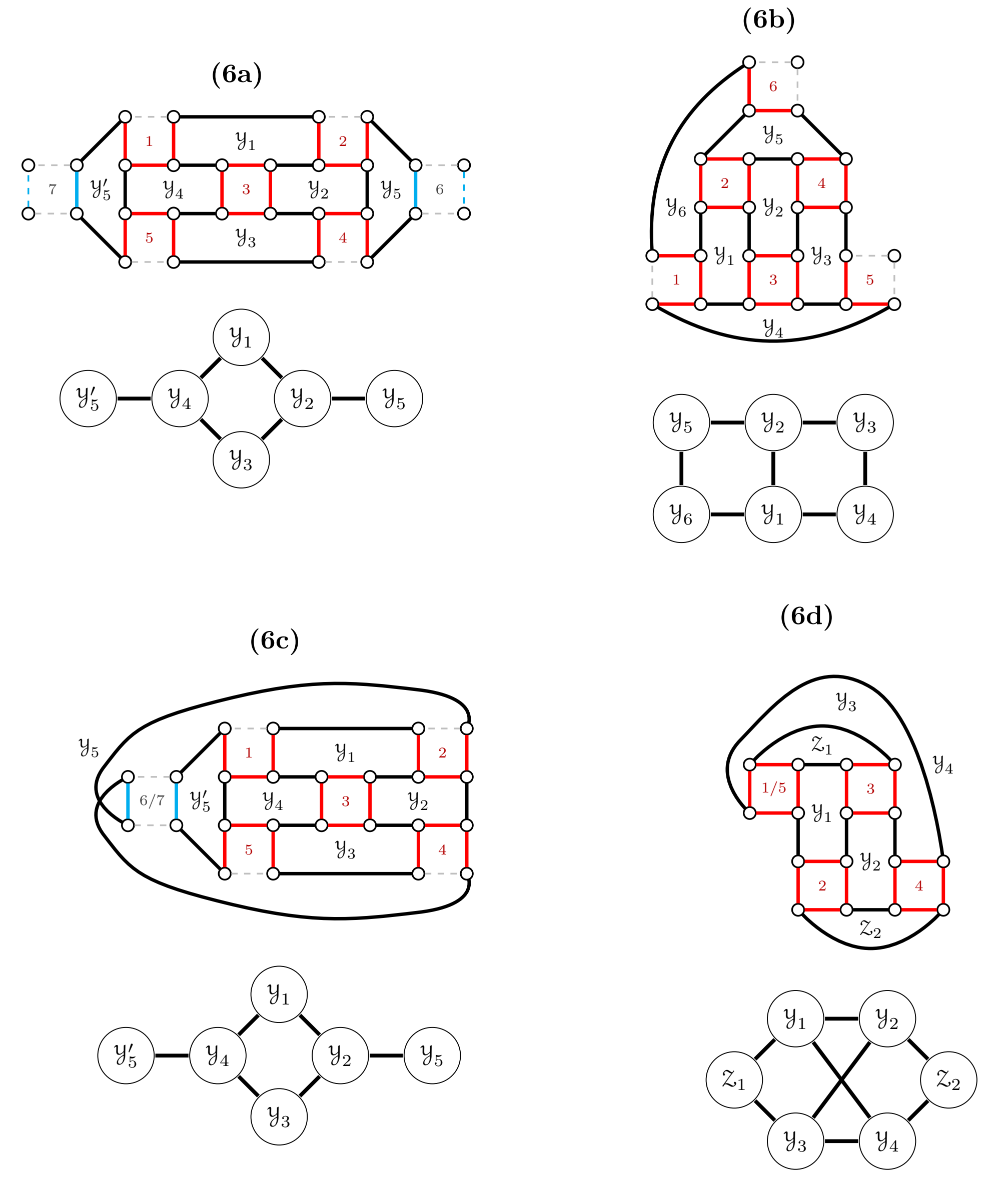

Figure S5 Bubbles with six cycles. (6a) is derived from (5a) by adding a $\check{\mathbb{D}}\mathbb{S}\check{\mathbb{D}}$-path and (6b) is derived from (5a) by adding a $\check{\mathbb{D}}$-edge. (6c) is derived from (6a) by merging squares. (6d) is obtained by connecting the two free vertices of (4e), adding a 4- and a 6-cycle.

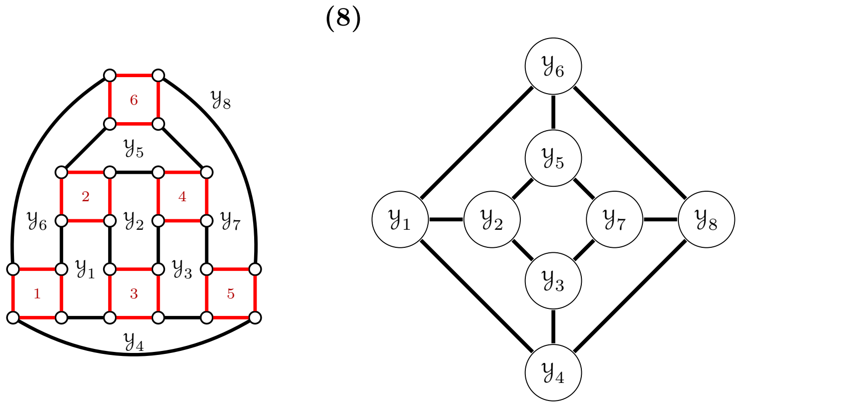

Figure S6 Single case of a bubble with eight cycles, obtained by connecting the two free vertices of (6b). All squares are fully connected and the bubble can no longer be extended.

(A).2 Balanced cycle-bubbles intersecting with more than one path

In Figures A7-A9 we enumerate all cases of balanced cycle-bubbles that have at most 8 cycles and intersect with more than one path. We omit the general and well described case of a cycle-line with plug connections. In all figures, dotted red edges are exclusively for paths, dashed gray edges are pruned out, blue nodes represent $\mathbb{S}$-telomeres and gray nodes represent $\check{\mathbb{D}}$-telomeres. Furthermore, green/yellow solutions are co-optimal, while yellow solutions are better than the pink alternatives.

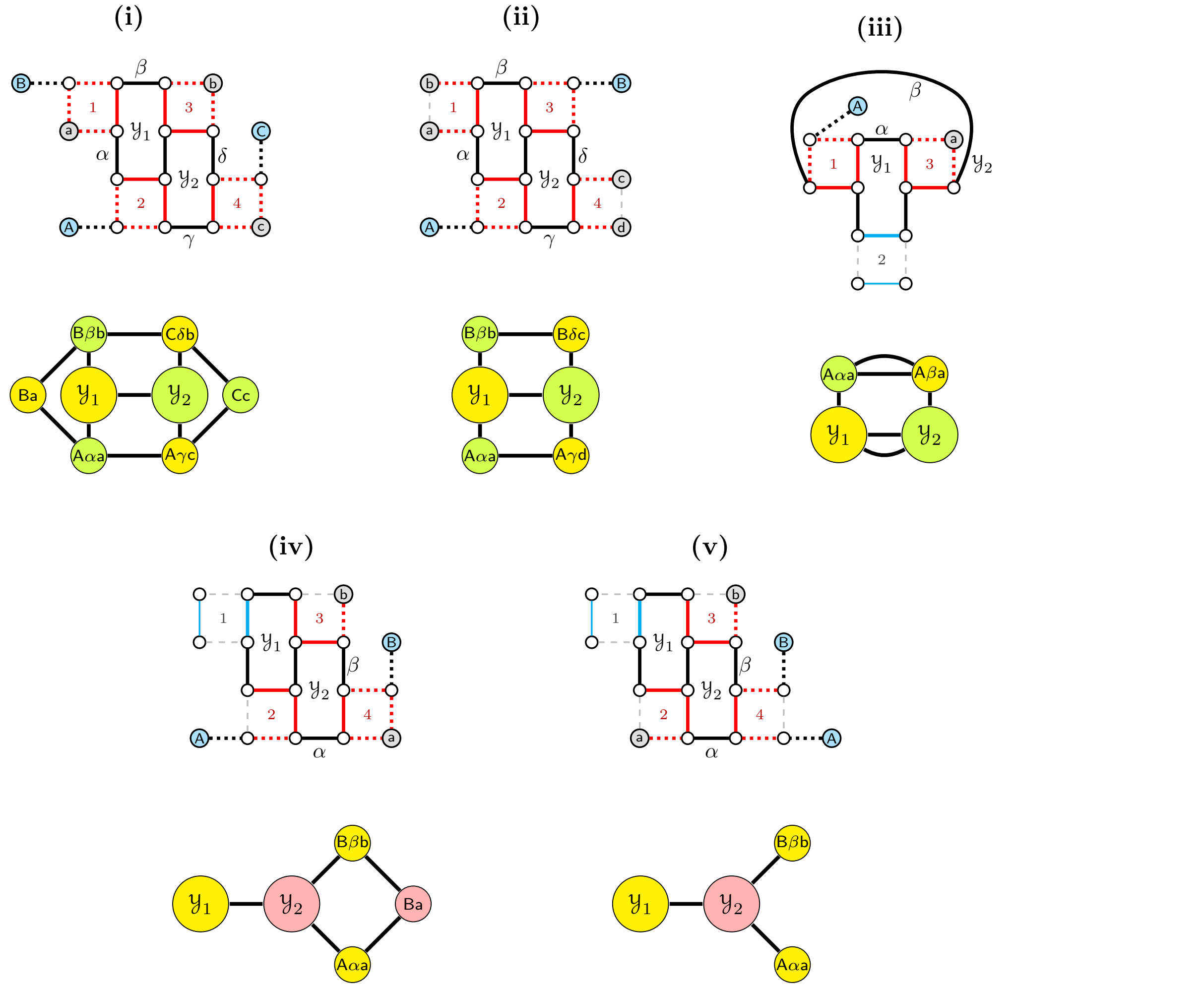

Figure S7 Bubbles with two 6-cycles $\mathcal{Y}_1$ and $\mathcal{Y}_2$ (solid edges) whose intersections with paths do not allow path-line extensions. (i) is symmetrically surrounded by a cyclic path-line of length 6. (ii) is symmetrically connected to two path-lines of length 2. (iii) is symmetrically connected to a single path-line of length 2, whose paths intersect at both telomeres. (iv) has a path line of length 3, whose ends are connected to a single cycle. (v) has two paths connected to a single cycle.

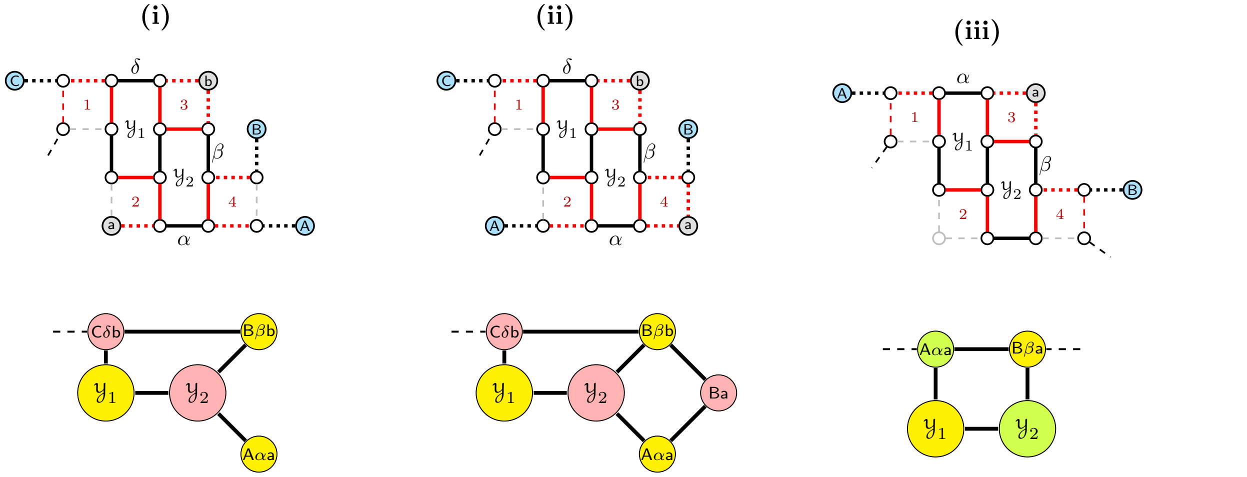

Figure S8 Bubbles with two 6-cycles y_1 and y_2 (solid edges) whose intersections with paths allows at least one path-line extension. (i) has a path and an extendable path-line, the single path and the first vertex of the path-line are connected to one cycle, while the second vertex of the path-line is connected to the other cycle. (ii) has a single extendable path-line whose first and third paths are connected to one cycle, while the fourth path is connected to the other cycle. (iii) is symmetrically connected to a single path-line that can be extended in both directions.

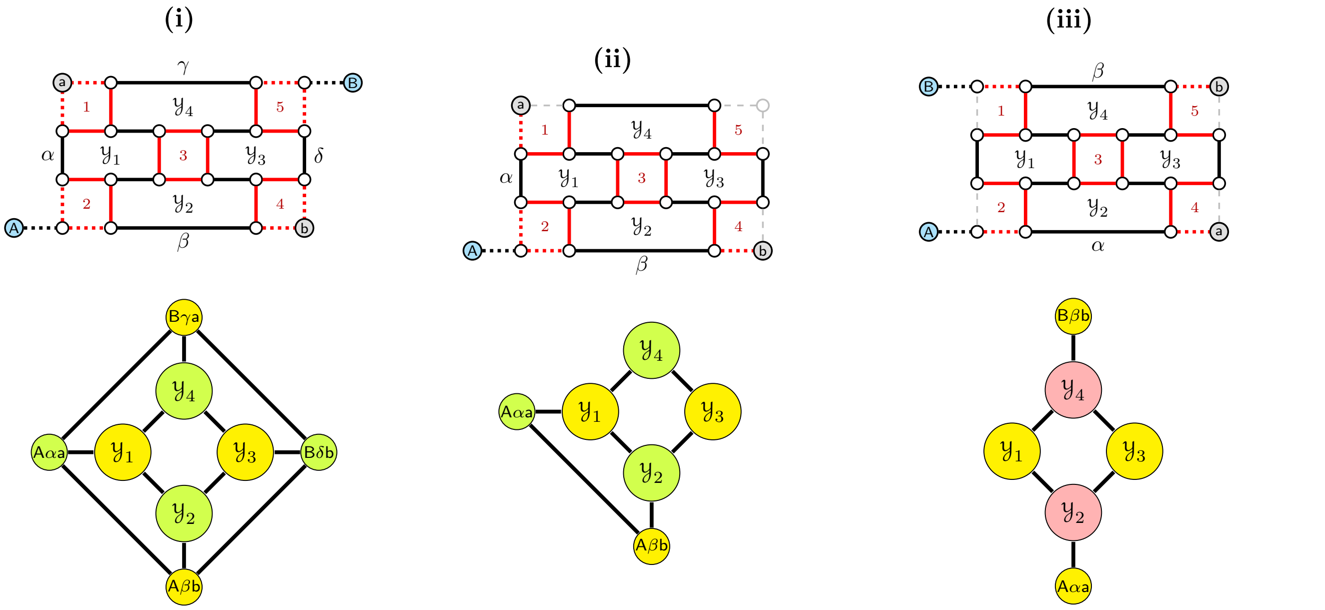

Figure S9 All possibilities for a bubble of four 6-cycles with intersecting paths. (i) is symmetrically surrounded by a cyclic path-line of length 4. (ii) has two paths forming a path line and two connections between these paths and cycles from distinct independent sets. (iii) has has two paths and two connections between these paths and cycles from the same independent set.
